# Case report: Delayed cardiac rupture with congenital absence of pericardium after blunt trauma

**DOI:** 10.3389/fcvm.2022.1079670

**Published:** 2022-12-20

**Authors:** Tuo Shen, He Fang, Tao Tang, Hongtai Tang, Xiaoyan Hu, Feng Zhu

**Affiliations:** Burns and Trauma ICU, The First Affiliated Hospital, Naval Medical University, Shanghai, China

**Keywords:** congenital absence of pericardium, delayed cardiac rupture, myocardial contusion, blunt trauma, chest trauma, case report

## Abstract

A 66 years old male was admitted to our hospital after a serious car accident. The patient presented with severe shock after admission. After the examination, the patient was diagnosed with hemopneumothorax and myocardial contusion, accompanied by spleen rupture. After emergency surgery and a series of symptomatic treatments, the patient’s condition gradually stabilized. One week later, the patient suddenly presented with severe shock. Massive hemothorax was found on the left side of the chest. Surgical exploration revealed cardiac rupture and accidental absence of congenital pericardium. According to the literature review, congenital absence of pericardium (CAP) is relatively rare. Although there are certain imaging features, the clinical diagnosis is very difficult. However, this patient did not show the characteristics in the literature and had some other atypical features. The role of CAP in the occurrence and development of the patient’s heart injury and rupture is worthy of discussion. What we learned from this case is that we should look for potential risks in the telltale signs of a patient’s condition.

## Introduction

**FIGURE 1:**
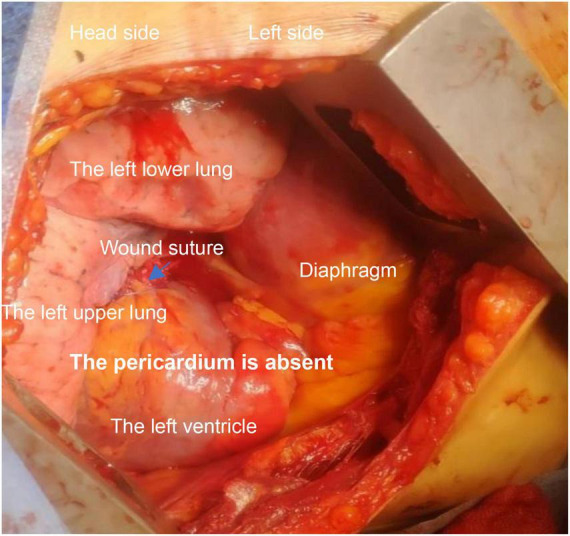
Intraoperative observations: right side decubitus position; left heart exposed; left side posterior wall rupture has been sutured; there is still continuous bleeding.

Congenital absence of pericardium (CAP) (see Figure 1) is rare in clinical practice (1). It includes complete and partial pericardial absence, and left pericardial absence is common. The absence of pericardium often lacks typical imaging manifestations and clinical signs, and some patients may present with shortness of breath, chest pain, dizziness, and other discomfort. Some patients may die suddenly of unknown causes, and some patients may be accidentally diagnosed during surgeries (2).

Chest trauma is the third most common cause of trauma. Chest trauma has a high morbidity and mortality, associated with 25% of trauma-related deaths (3). The incidence of cardiac contusion in blunt chest trauma ranges from 3 to 56% (4). Blunt cardiac rupture is a very rare condition with a high mortality rate, and the incidence of blunt cardiac rupture is 1/2,400 in the United States (5). Delayed blunt cardiac rupture is less common clinically.

Congenital absence of pericardium was incidentally discovered in the treatment of a patient with delayed blunt cardiac rupture.

## Case presentation

**FIGURE 2:**
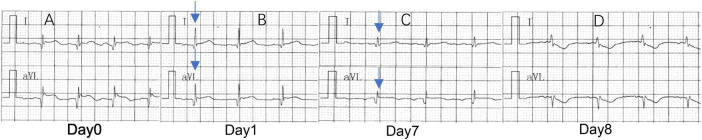
**(A)** Sinus rhythm, abnormal Q-wave and ST-segment elevation on I and aVL, suggesting acute high lateral myocardial infarction. **(B)** Sinus rhythm, abnormal Q-waves on I and aVL, suggesting high lateral myocardial infarction. **(C)** Sinus rhythm, no obvious abnormality on I and aVL. **(D)** Sinus tachycardia, ST-segment depression on I and aVL, suggesting increased myocardial ischemia. Analyses the ECG results, 1 day before cardiac rupture, QRS voltage on I and aVL was significantly lower.

**FIGURE 3:**
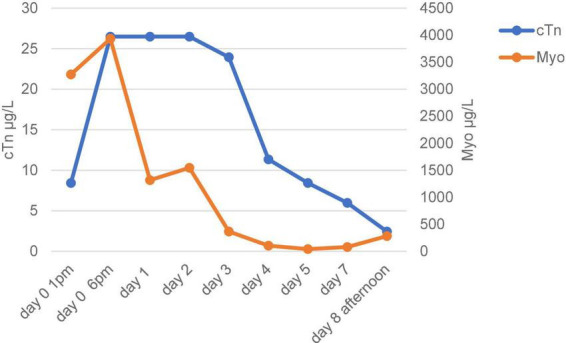
Time courses of troponin (cTn) and myoglobin (Myo).

**FIGURE 4:**
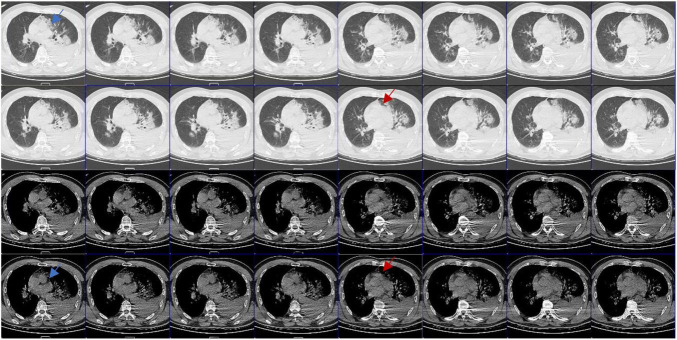
Computed tomography (CT) images in different window settings: As the blue arrow shows, there is no obvious pericardial structure in the left area of pulmonary artery, which is closely related to lung tissue and has irregular edge; as the red arrow shows, there is an abnormal notch near the pulmonary artery, which is rare when the pericardium is intact.

**FIGURE 5:**
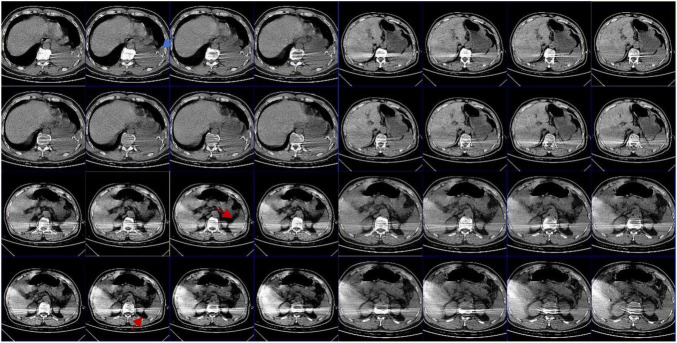
Abdominal CT: the blue arrow shows intrathoracic drain; the red arrow shows that there appears to be exudate around the spleen; the splenic hilum appears to be intact.

**FIGURE 6:**
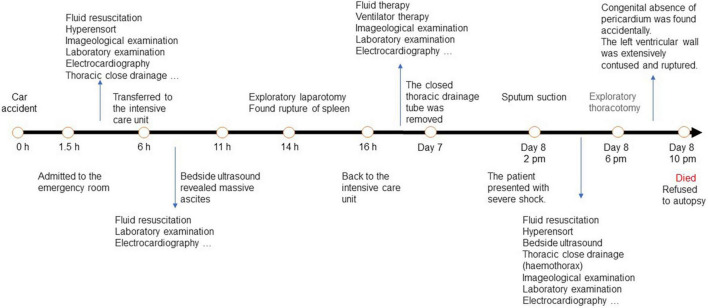
Timeline.

A 66 years old male was admitted to the hospital due to a rear-end crash, in which case, the patient in the back seat was squeezed in the chest by the front seat. The patient complained of multiple body pain with chest tightness and shortness of breath. He had no previous history of coronary heart disease, angina pectoris or myocardial infarction. The patient developed severe shock in the emergency room with a blood pressure of 78/40 mmHg. The electrocardiogram (ECG) indicated a high wall myocardial infarction (see Figure 2). The troponin was 8.434 μg/L (see Figure 3). Computed tomography (CT) indicated “multiple rib fracture on the left side, hydropneumothorax on the left side, no obvious abnormality in the skull and abdomen”(see Figures 4, 5). Closed thoracic drainage was given. The drainage volume was reduced and stabilized after drainage of 1,300 ml bloody fluid. Resuscitation with adequate fluids and high doses of vasoactive drugs, however, is not effective. Bedside ultrasonography indicated a large amount of ascites, and the abdominocentesis got blood that doesn’t clot. Emergency laparotomy revealed splenic rupture and contusion of pancreatic tail, and splenectomy was performed. After symptomatic treatment, the circulation was gradually stabilized. One week later, the thoracic drainage volume was 40 ml for 2 consecutive days, and the thoracic drainage tube was removed. The next day, however, the patient was suddenly agitated and sweating profuse, followed by no response and weak pulsation of the aorta. Resuscitation was initiated Immediately, meanwhile, bedside ultrasound showed a large pleural effusion on the left side. Closed thoracic drainage was performed to drain a large amount of blood continuously, and exploratory thoracotomy was performed in emergency. Intraoperative exploration revealed massive hematocele and blood clots in the thoracic cavity. After the removal of the hematocele and clots, the left heart was bare and the pericardium was absent (see Figure 1). We found widespread contusion on the lateral posterior wall of the left ventricle with a 5mm laceration where was sustained bleeding. The bleeding was stopped by pressing, and the blood pressure was controlled before suture and repair. Severe and extensive contusion and necrosis of myocardial tissue resulted in failure of repair and death. The figure below shows a timeline of the patient’s medical history (see Figure 6).

## Discussion

Congenital absence of pericardium is often difficult to diagnose, and it has no specific symptoms and signs, so it is often ignored. CAP has certain imaging diagnostic features (6, 7): (1) pulmonary parenchyma between the main pulmonary artery and the ascending aorta; (2) pulmonary parenchyma between the base of the heart and the left diaphragmatic muscle; (3) pulmonary parenchyma between the ascending main aorta and the right pulmonary artery; (4) “SNOOPY” sign on chest radiograph. Even so, it is still very difficult to clinically diagnose CAP. Sergio et al. (6), reviewed the imaging examinations of 12,888 patients and found only 1 case with partial pericardial defect (left heart) with conditions (1) and (2), and 10 cases with false positive results. Reviewing the radiographic findings of this patient after the injury, we did find some evidence of CAP (see Figure 4), but did not find any obvious radiographic features in accordance with the literature.

Computed tomography and magnetic resonance imaging (MRI) are the gold standard for the diagnosis of CAP (7), but it is still difficult to make a diagnosis in the absence of symptoms and experience, so that in many cases the diagnosis is made during surgeries. In this case, CAP was missed by CT. Due to severe lung contusion, the patient was dependent on ventilator support. We didn’t have the opportunity to perform MRI or any other examinations.

The pericardium supports and protects the heart, limiting its expansion, cushioning the impact of violent heart injury, mitigating the injury, and preventing the heart from rapidly rupturing when pressure rises.

Due to the lack of pericardial protection, after a violent injury to the chest, the myocardium may suffer more severe contusion under the compression of the front and rear chest wall structures. Shah et al. (8), suggested that cardiac injury should be highly suspected in any patient with blunt chest trauma and multiple injuries, and that baseline chest radiographs, ECG, and cardiac enzyme measurements should be part of the routine evaluation of these patients to rule out cardiac injury, and echocardiography should be performed if clinically necessary. The imaging manifestations of blunt myocardial contusion are atypical, and the electrocardiographic findings are sometimes indistinguishable from myocardial infarction. In this patient, the early ECG showed a high lateral wall myocardial infarction and his myocardial enzymes increased significantly. Excluding the history of coronary heart disease and myocardial infarction, the diagnosis of cardiac contusion was relatively accurate in combination with the mechanism of injury and the extensive hemothorax and multiple rib fractures. But it was hard to diagnose an acute heart rupture. In the presence of the pericardium, cardiac rupture may be accompanied by pericardium tamponade. Most patients died at the scene or in the early stages of rescue. In the blunt injury, simultaneous rupture of the heart and pericardium is rare. Coincidentally, this patient happened to have no pericardium. A heart rupture at the time of the injury can be very difficult to determine. The small rupture in the heart may have closed during the patient’s hypotension. Even if that was what really happened, we could not diagnose acute cardiac rupture due to the lack of effective early evaluation methods. Moreover, due to the complexity of the condition, no further evaluation was performed in the case of no significant change in treatment strategy. Meanwhile, combined with the Flotrac/Vigileo system, the patient’s cardiac function was relatively stable 1 week after the injury. As the patient’s condition improved, the cardiac preload and afterload increased. But the damaged myocardium gradually dissolved. The ventricular wall became weak. The patients’ intolerance to endotracheal intubation and airway response to stimulation such as sputum aspiration after recovery will lead to fluctuations in blood pressure, which increased the burden on the left heart imperceptibly. Without the restriction and protection of the pericardium, the blunt heart was more likely to rupture. During the final cardiac surgery, we found extensive bruising of the myocardium surrounding the rupture. This suggests that the heart rupture may be secondary to myocardial contusion. Unfortunately, we did not have the opportunity to conduct autopsy or pathological examination after the operation.

The patient’s condition changed suddenly, progressed rapidly, and the clinical diagnosis was not clear. Generally, patients with heart rupture often die of tamponade at an early stage. Due to the absence of the pericardium, the patient showed symptoms of heart failure and shock during the process of heart rupture. After a large amount of transfusion and fluid rehydration, there was still a chance to perform thoracotomy exploration, which to a certain extent created a rescue opportunity for clinicians.

Our early assessment for cardiac contusion was flawed in this patient. Evaluation by echocardiography was necessary. We can evaluate the motion of the ventricular wall and observe the pericardial effusion. By chance, maybe, we could find the CAP. Unfortunately, the emergency room and intensive care unit were not equipped to perform echocardiography.

The patient still showed signs of the onset of a ruptured heart. ECG changes before and after the onset still had a certain suggestive effect (see Figure 2). The patient’s I lead and aVL leads showed significant amplitude changes the day before the onset of the disease. Dynamic comparison of ECG changes could enable us to warn of the potential risk of heart rupture in advance and make adequate clinical response measures.

## Conclusion

1. The clinical diagnosis of CAP is very difficult. Routine imaging examinations sometimes lack specificity. Therefore, the diagnosis should be made with the help of clinical experience and targeted tests.
2. Without the protective effect of the pericardium, the heart is more vulnerable to injury and more likely to cause serious adverse events in both complete and partial pericardial absence during chest trauma.
3. In the treatment of patients with chest trauma, the presence of cardiac contusion should be vigilant. For patients with severe cardiac contusion, there is still a risk of delayed rupture even in about 1 week after injury. Therefore, it is necessary to systematically evaluate the severity of cardiac contusion.
4. In the rescue stage of patients with severe chest trauma, we can use ECG, echocardiography, chest CT and laboratory tests to evaluate cardiopulmonary injuries. For patients with cardiac contusion, it is still necessary to regularly evaluate with the help of various methods in the subsequent treatment. Such as MRI, coronary angiography, etc.
5. We should observe the change process of auxiliary examination dynamically, especially the change of some indexes closely related to injury.

## Data availability statement

The original contributions presented in this study are included in the article/Supplementary material, further inquiries can be directed to the corresponding author.

## Ethics statement

Written informed consent was obtained from the individual(s) for the publication of any potentially identifiable images or data included in this article.

## Author contributions

FZ came up with the idea. TS drafted the manuscript. HF and TT collected and summarized the medical records. HT, XH, and FZ revised the manuscript. All authors read and approved the final manuscript.
