# Supplementary material for: Case report: Delayed cardiac rupture with congenital absence of pericardium after blunt trauma
Source: Front Cardiovasc Med. 2022 Dec 20;9:1079670. doi: 10.3389/fcvm.2022.1079670 (PMC9808968; doi:10.3389/fcvm.2022.1079670)
Supplement: Supplementary file 1 [file Data_Sheet_1.pdf]

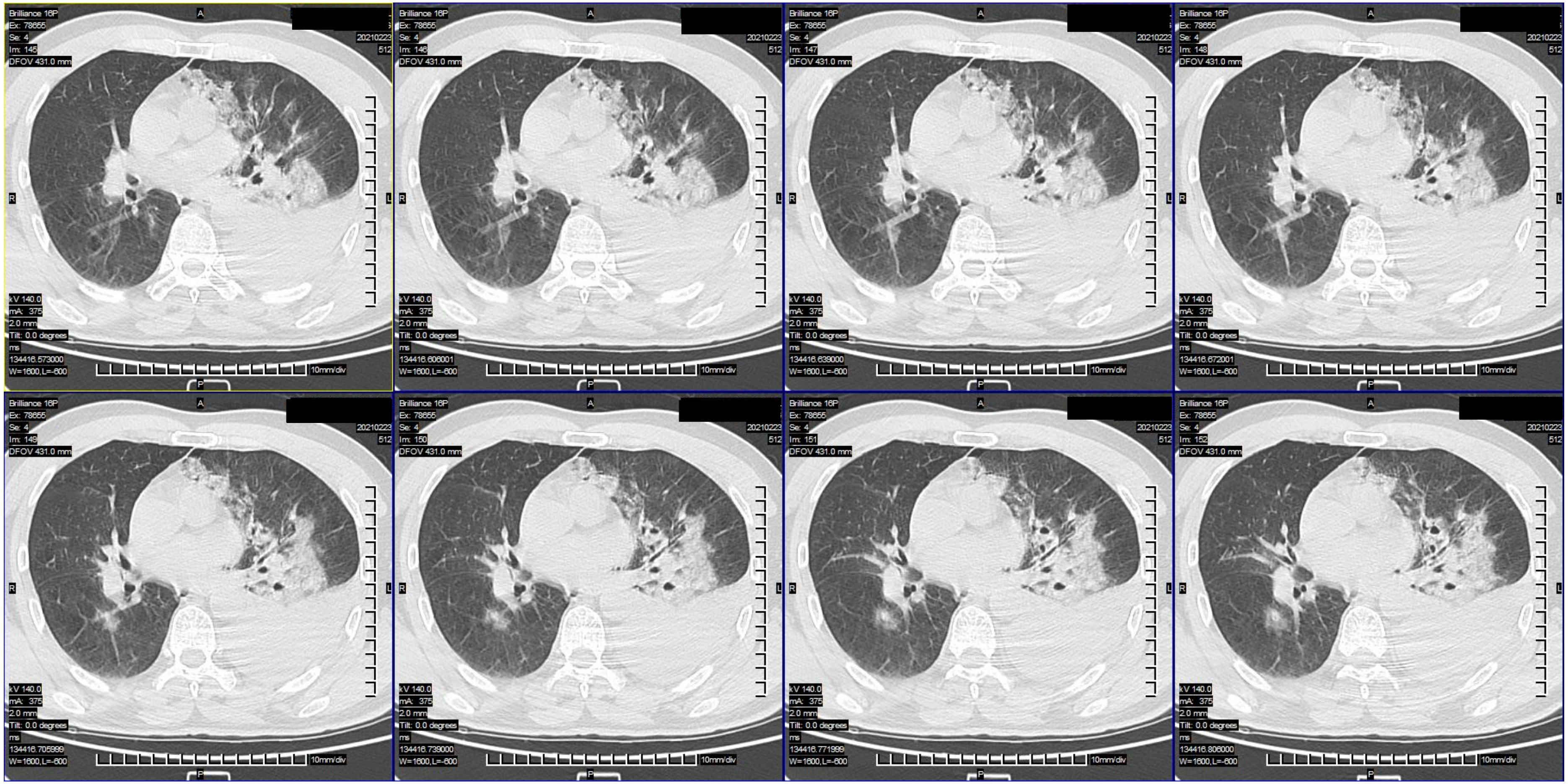
Brilliance 16P
A
Ex: 78655
Se: 4
Im: 145
DFOV 431.0 mm
[redacted]
20210223
512
R
L
kV 140.0
mA: 375
2.0 mm
Tilt: 0.0 degrees
ms
134416.573000
W=1600,L=-600
10mm/div
P
Brilliance 16P
A
Ex: 78655
Se: 4
Im: 146
DFOV 431.0 mm
[redacted]
20210223
512
R
L
kV 140.0
mA: 375
2.0 mm
Tilt: 0.0 degrees
ms
134416.606001
W=1600,L=-600
10mm/div
P
Brilliance 16P
A
Ex: 78655
Se: 4
Im: 147
DFOV 431.0 mm
[redacted]
20210223
512
R
L
kV 140.0
mA: 375
2.0 mm
Tilt: 0.0 degrees
ms
134416.639000
W=1600,L=-600
10mm/div
P
Brilliance 16P
A
Ex: 78655
Se: 4
Im: 148
DFOV 431.0 mm
[redacted]
20210223
512
R
L
kV 140.0
mA: 375
2.0 mm
Tilt: 0.0 degrees
ms
134416.672001
W=1600,L=-600
10mm/div
P
Brilliance 16P
A
Ex: 78655
Se: 4
Im: 149
DFOV 431.0 mm
[redacted]
20210223
512
R
L
kV 140.0
mA: 375
2.0 mm
Tilt: 0.0 degrees
ms
134416.705999
W=1600,L=-600
10mm/div
P
Brilliance 16P
A
Ex: 78655
Se: 4
Im: 150
DFOV 431.0 mm
[redacted]
20210223
512
R
L
kV 140.0
mA: 375
2.0 mm
Tilt: 0.0 degrees
ms
134416.739000
W=1600,L=-600
10mm/div
P
Brilliance 16P
A
Ex: 78655
Se: 4
Im: 151
DFOV 431.0 mm
[redacted]
20210223
512
R
L
kV 140.0
mA: 375
2.0 mm
Tilt: 0.0 degrees
ms
134416.771999
W=1600,L=-600
10mm/div
P
Brilliance 16P
A
Ex: 78655
Se: 4
Im: 152
DFOV 431.0 mm
[redacted]
20210223
512
R
L
kV 140.0
mA: 375
2.0 mm
Tilt: 0.0 degrees
ms
134416.806000
W=1600,L=-600
10mm/div
P

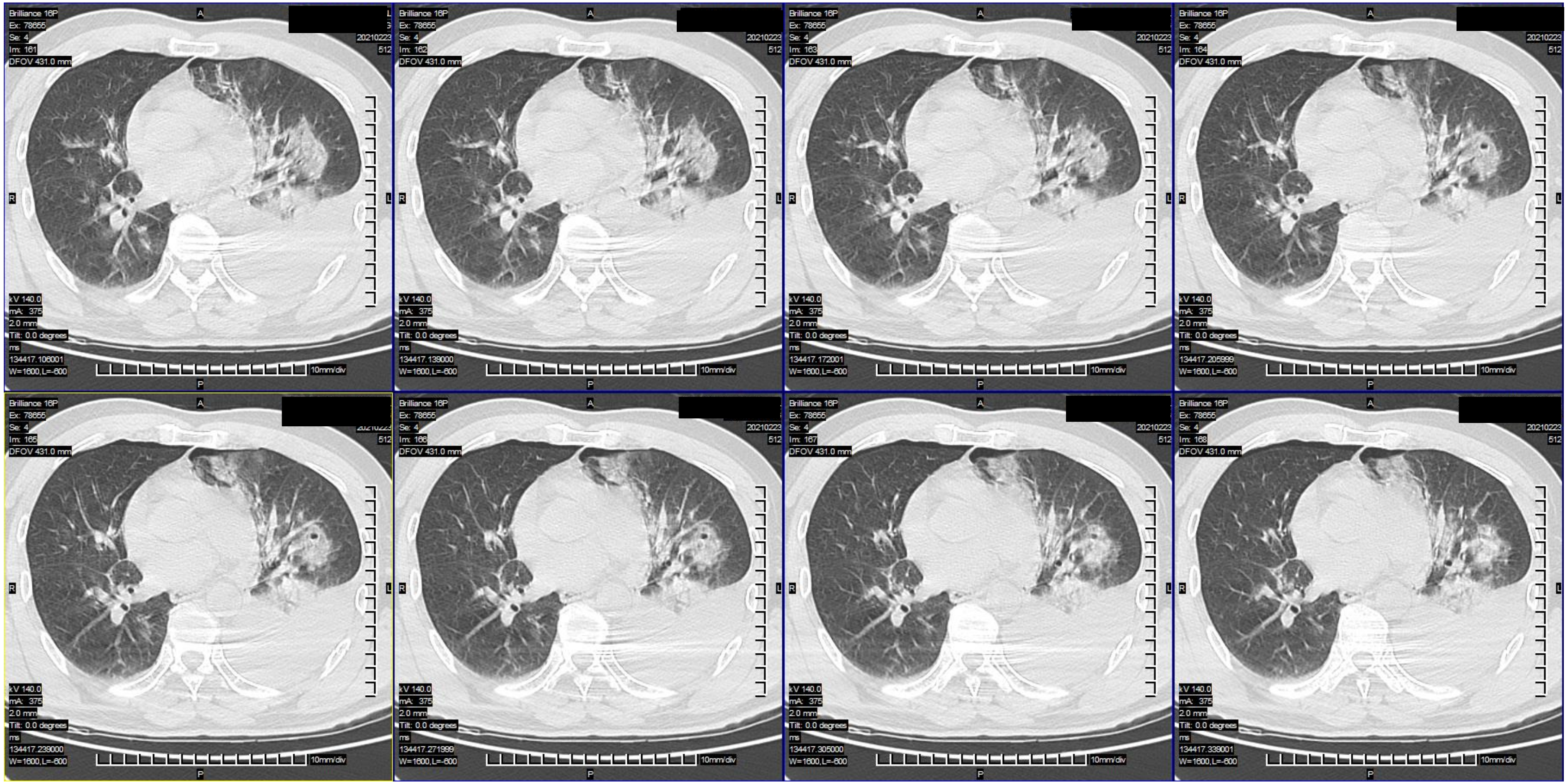

Brilliance 16P
Ex: 78655
Se: 4
Im: 161
DFOV 431.0 mm
[redacted]
20210223
512
A
R
L
P
kV 140.0
mA: 375
2.0 mm
Tilt: 0.0 degrees
ms
134417.106001
W=1600,L=-600
10mm/div
Brilliance 16P
Ex: 78655
Se: 4
Im: 162
DFOV 431.0 mm
[redacted]
20210223
512
kV 140.0
mA: 375
2.0 mm
Tilt: 0.0 degrees
ms
134417.139000
W=1600,L=-600
10mm/div
Brilliance 16P
Ex: 78655
Se: 4
Im: 163
DFOV 431.0 mm
[redacted]
20210223
512
kV 140.0
mA: 375
2.0 mm
Tilt: 0.0 degrees
ms
134417.172001
W=1600,L=-600
10mm/div
Brilliance 16P
Ex: 78655
Se: 4
Im: 164
DFOV 431.0 mm
[redacted]
20210223
512
kV 140.0
mA: 375
2.0 mm
Tilt: 0.0 degrees
ms
134417.205999
W=1600,L=-600
10mm/div
Brilliance 16P
Ex: 78655
Se: 4
Im: 165
DFOV 431.0 mm
[redacted]
512
kV 140.0
mA: 375
2.0 mm
Tilt: 0.0 degrees
ms
134417.239000
W=1600,L=-600
10mm/div
Brilliance 16P
Ex: 78655
Se: 4
Im: 166
DFOV 431.0 mm
[redacted]
20210223
512
kV 140.0
mA: 375
2.0 mm
Tilt: 0.0 degrees
ms
134417.271999
W=1600,L=-600
10mm/div
Brilliance 16P
Ex: 78655
Se: 4
Im: 167
DFOV 431.0 mm
[redacted]
20210223
512
kV 140.0
mA: 375
2.0 mm
Tilt: 0.0 degrees
ms
134417.305000
W=1600,L=-600
10mm/div
Brilliance 16P
Ex: 78655
Se: 4
Im: 168
DFOV 431.0 mm
[redacted]
20210223
512
kV 140.0
mA: 375
2.0 mm
Tilt: 0.0 degrees
ms
134417.339001
W=1600,L=-600
10mm/div

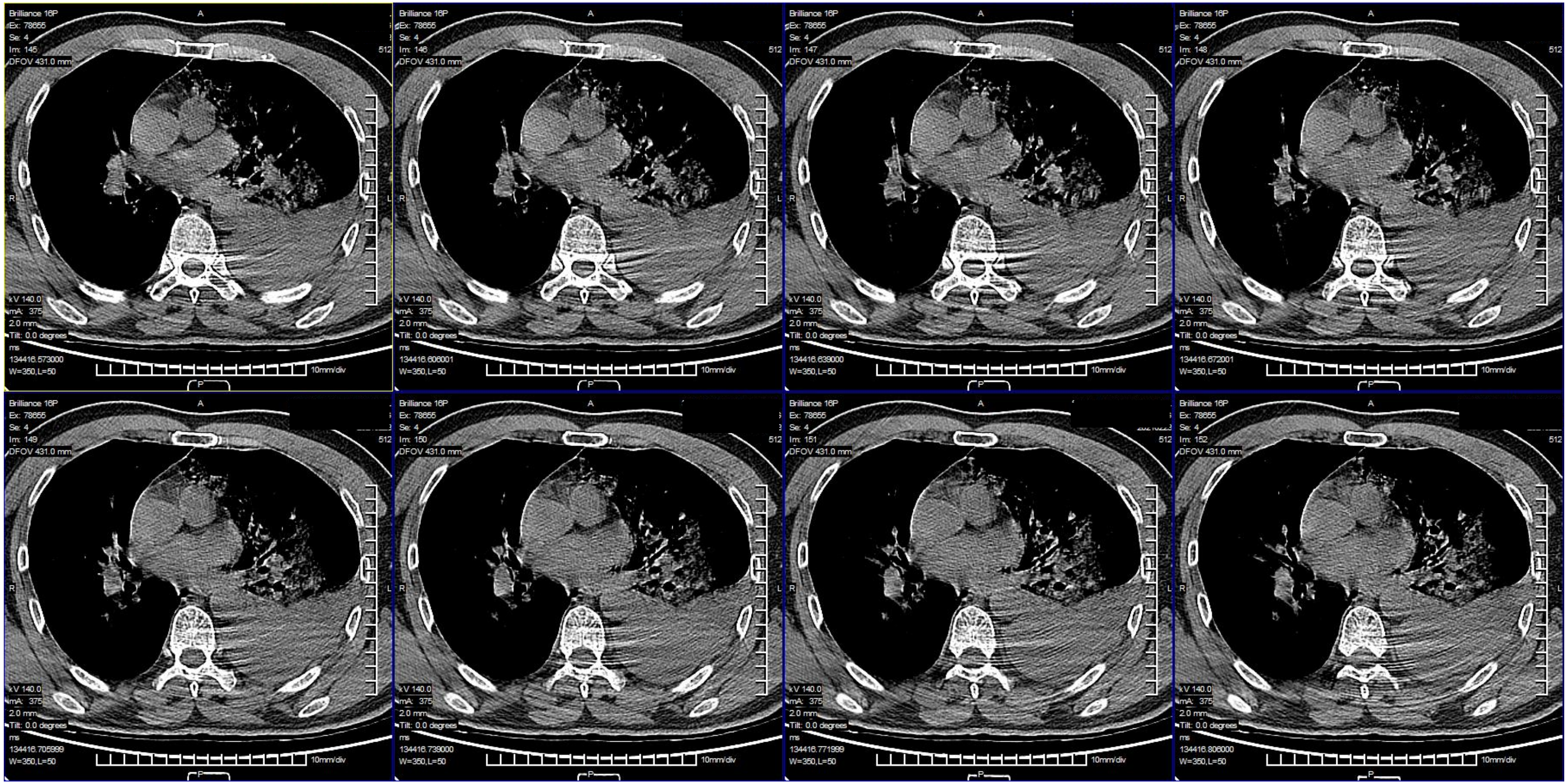
Brilliance 16P
A
Ex: 78655
Se: 4
Im: 145
DFOV 431.0 mm
512
R
L
kV 140.0
mA: 375
2.0 mm
Tilt: 0.0 degrees
ms
134416.573000
W=350,L=50
10mm/div
P
Brilliance 16P
A
Ex: 78655
Se: 4
Im: 146
DFOV 431.0 mm
512
R
L
kV 140.0
mA: 375
2.0 mm
Tilt: 0.0 degrees
ms
134416.606001
W=350,L=50
10mm/div
P
Brilliance 16P
A
Ex: 78655
Se: 4
Im: 147
DFOV 431.0 mm
512
R
L
kV 140.0
mA: 375
2.0 mm
Tilt: 0.0 degrees
ms
134416.639000
W=350,L=50
10mm/div
P
Brilliance 16P
A
Ex: 78655
Se: 4
Im: 148
DFOV 431.0 mm
512
R
L
kV 140.0
mA: 375
2.0 mm
Tilt: 0.0 degrees
ms
134416.672001
W=350,L=50
10mm/div
P
Brilliance 16P
A
Ex: 78655
Se: 4
Im: 149
DFOV 431.0 mm
512
R
L
kV 140.0
mA: 375
2.0 mm
Tilt: 0.0 degrees
ms
134416.705999
W=350,L=50
10mm/div
P
Brilliance 16P
A
Ex: 78655
Se: 4
Im: 150
DFOV 431.0 mm
512
R
L
kV 140.0
mA: 375
2.0 mm
Tilt: 0.0 degrees
ms
134416.739000
W=350,L=50
10mm/div
P
Brilliance 16P
A
Ex: 78655
Se: 4
Im: 151
DFOV 431.0 mm
512
R
L
kV 140.0
mA: 375
2.0 mm
Tilt: 0.0 degrees
ms
134416.771999
W=350,L=50
10mm/div
P
Brilliance 16P
A
Ex: 78655
Se: 4
Im: 152
DFOV 431.0 mm
512
R
L
kV 140.0
mA: 375
2.0 mm
Tilt: 0.0 degrees
ms
134416.806000
W=350,L=50
10mm/div
P

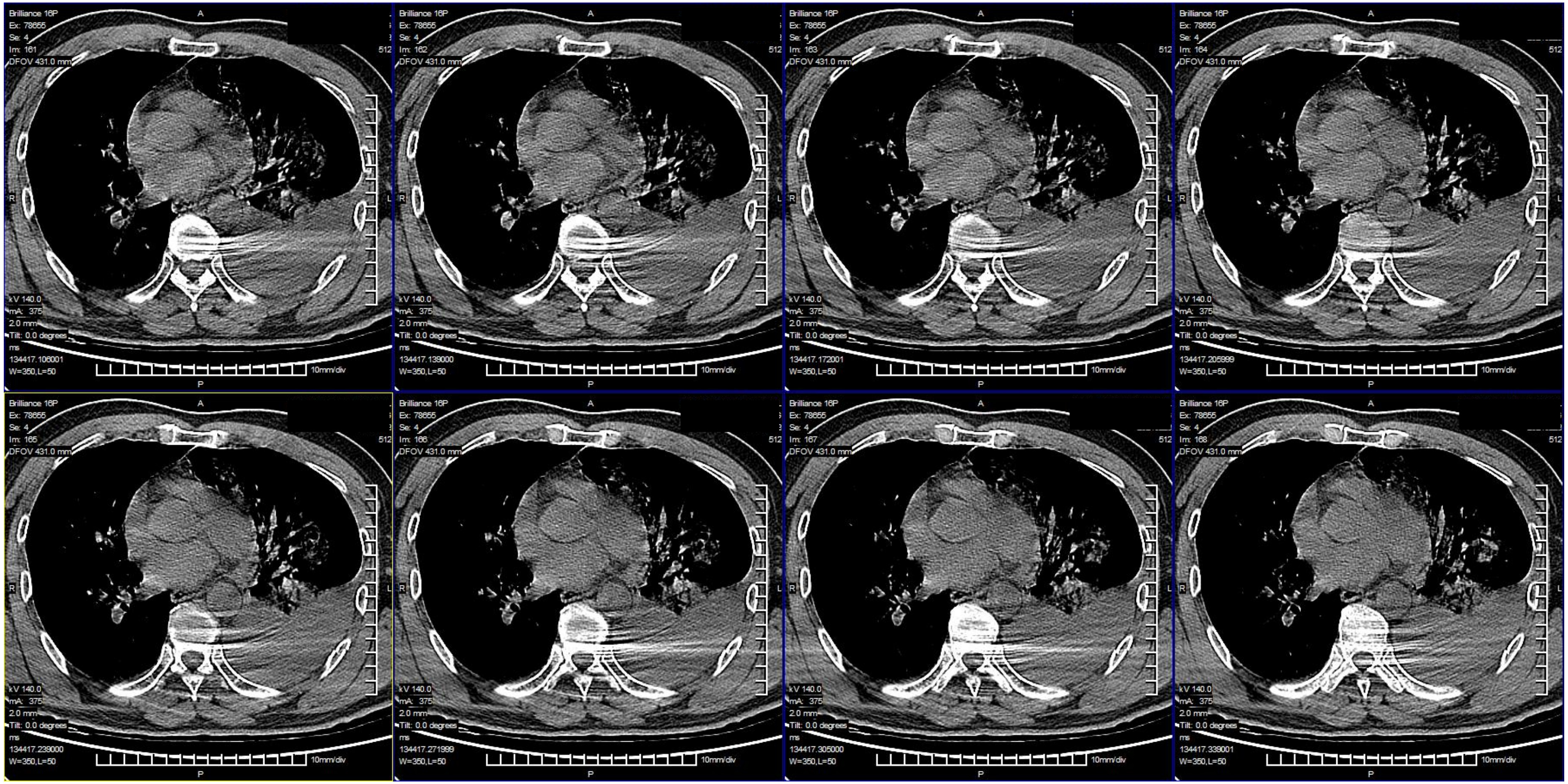
Brilliance 16P
A
Ex: 78655
Se: 4
Im: 161
DFOV 431.0 mm
512
R
L
kV 140.0
mA: 375
2.0 mm
Tilt: 0.0 degrees
ms
134417.106001
W=350,L=50
10mm/div
P
Brilliance 16P
A
Ex: 78655
Se: 4
Im: 162
DFOV 431.0 mm
512
R
L
kV 140.0
mA: 375
2.0 mm
Tilt: 0.0 degrees
ms
134417.139000
W=350,L=50
10mm/div
P
Brilliance 16P
A
Ex: 78655
Se: 4
Im: 163
DFOV 431.0 mm
512
R
L
kV 140.0
mA: 375
2.0 mm
Tilt: 0.0 degrees
ms
134417.172001
W=350,L=50
10mm/div
P
Brilliance 16P
A
Ex: 78655
Se: 4
Im: 164
DFOV 431.0 mm
512
R
L
kV 140.0
mA: 375
2.0 mm
Tilt: 0.0 degrees
ms
134417.205999
W=350,L=50
10mm/div
P
Brilliance 16P
A
Ex: 78655
Se: 4
Im: 165
DFOV 431.0 mm
512
R
L
kV 140.0
mA: 375
2.0 mm
Tilt: 0.0 degrees
ms
134417.239000
W=350,L=50
10mm/div
P
Brilliance 16P
A
Ex: 78655
Se: 4
Im: 166
DFOV 431.0 mm
512
R
L
kV 140.0
mA: 375
2.0 mm
Tilt: 0.0 degrees
ms
134417.271999
W=350,L=50
10mm/div
P
Brilliance 16P
A
Ex: 78655
Se: 4
Im: 167
DFOV 431.0 mm
512
R
L
kV 140.0
mA: 375
2.0 mm
Tilt: 0.0 degrees
ms
134417.305000
W=350,L=50
10mm/div
P
Brilliance 16P
A
Ex: 78655
Se: 4
Im: 168
DFOV 431.0 mm
512
R
L
kV 140.0
mA: 375
2.0 mm
Tilt: 0.0 degrees
ms
134417.339001
W=350,L=50
10mm/div
P

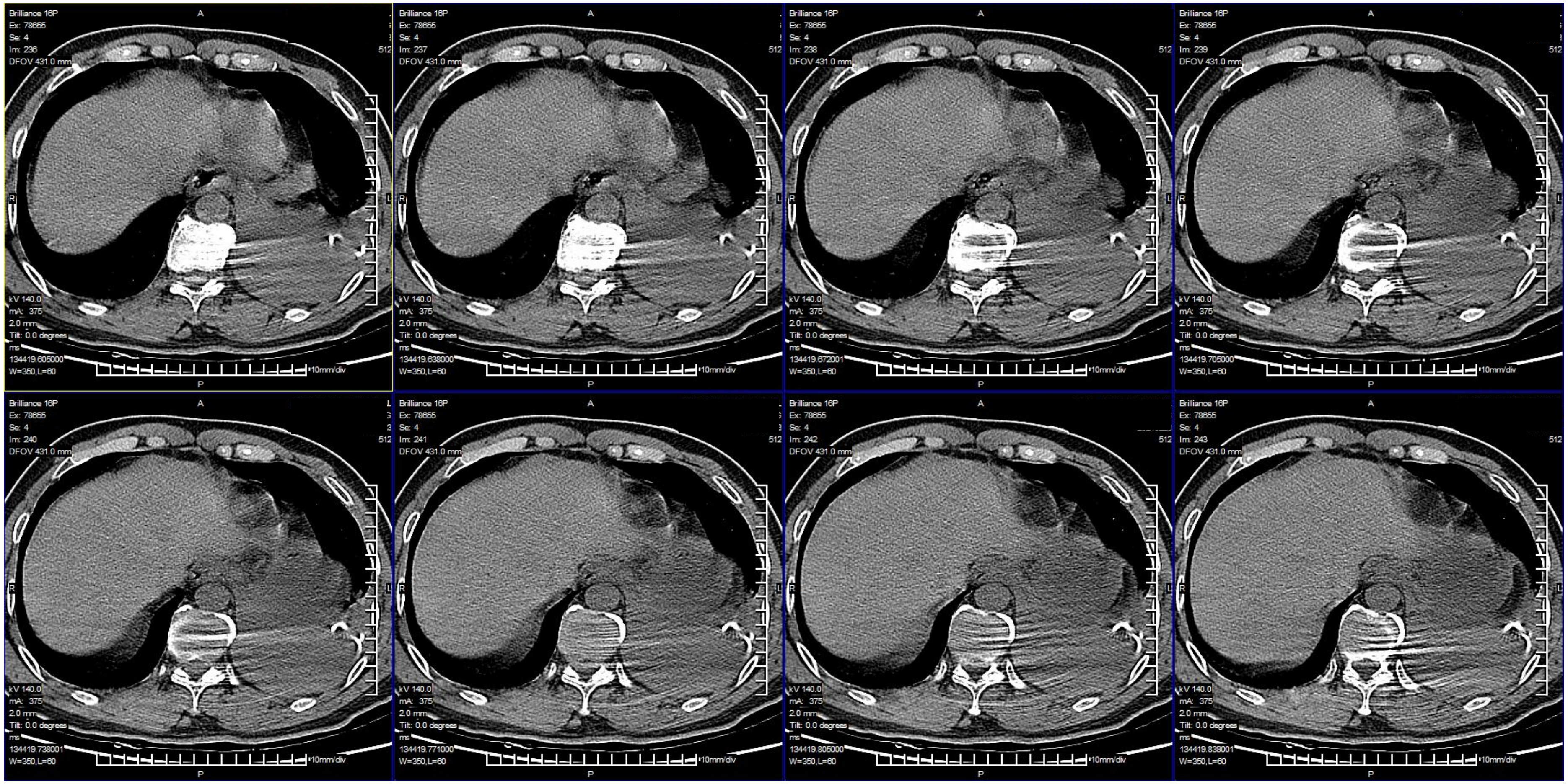
Brilliance 16P
Ex: 78655
Se: 4
Im: 236
DFOV 431.0 mm
kV 140.0
mA: 375
2.0 mm
Tilt: 0.0 degrees
ms
134419.605000
W=350,L=60
10mm/div
A
P
R
L
Brilliance 16P
Ex: 78655
Se: 4
Im: 237
DFOV 431.0 mm
kV 140.0
mA: 375
2.0 mm
Tilt: 0.0 degrees
ms
134419.638000
W=350,L=60
10mm/div
Brilliance 16P
Ex: 78655
Se: 4
Im: 238
DFOV 431.0 mm
kV 140.0
mA: 375
2.0 mm
Tilt: 0.0 degrees
ms
134419.672001
W=350,L=60
10mm/div
Brilliance 16P
Ex: 78655
Se: 4
Im: 239
DFOV 431.0 mm
kV 140.0
mA: 375
2.0 mm
Tilt: 0.0 degrees
ms
134419.705000
W=350,L=60
10mm/div
Brilliance 16P
Ex: 78655
Se: 4
Im: 240
DFOV 431.0 mm
kV 140.0
mA: 375
2.0 mm
Tilt: 0.0 degrees
ms
134419.738001
W=350,L=60
10mm/div
Brilliance 16P
Ex: 78655
Se: 4
Im: 241
DFOV 431.0 mm
kV 140.0
mA: 375
2.0 mm
Tilt: 0.0 degrees
ms
134419.771000
W=350,L=60
10mm/div
Brilliance 16P
Ex: 78655
Se: 4
Im: 242
DFOV 431.0 mm
kV 140.0
mA: 375
2.0 mm
Tilt: 0.0 degrees
ms
134419.805000
W=350,L=60
10mm/div
Brilliance 16P
Ex: 78655
Se: 4
Im: 243
DFOV 431.0 mm
kV 140.0
mA: 375
2.0 mm
Tilt: 0.0 degrees
ms
134419.839001
W=350,L=60
10mm/div

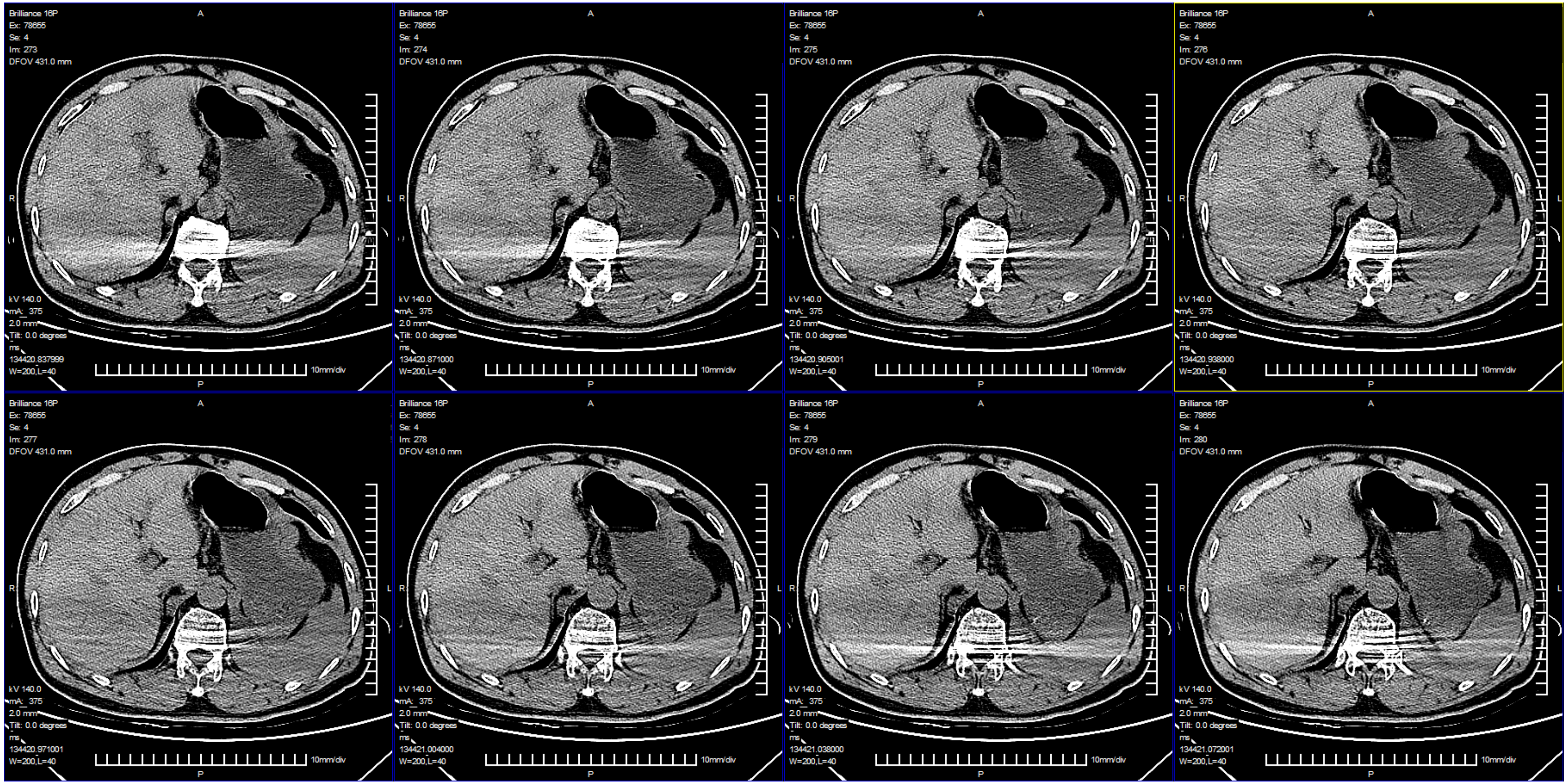
Brilliance 16P
Ex: 78655
Se: 4
Im: 273
DFOV 431.0 mm
A
R
L
kV 140.0
mA: 375
2.0 mm
Tilt: 0.0 degrees
ms
134420.837999
W=200,L=40
10mm/div
P
Brilliance 16P
Ex: 78655
Se: 4
Im: 274
DFOV 431.0 mm
A
R
L
kV 140.0
mA: 375
2.0 mm
Tilt: 0.0 degrees
ms
134420.871000
W=200,L=40
10mm/div
P
Brilliance 16P
Ex: 78655
Se: 4
Im: 275
DFOV 431.0 mm
A
R
L
kV 140.0
mA: 375
2.0 mm
Tilt: 0.0 degrees
ms
134420.905001
W=200,L=40
10mm/div
P
Brilliance 16P
Ex: 78655
Se: 4
Im: 276
DFOV 431.0 mm
A
R
L
kV 140.0
mA: 375
2.0 mm
Tilt: 0.0 degrees
ms
134420.938000
W=200,L=40
10mm/div
P
Brilliance 16P
Ex: 78655
Se: 4
Im: 277
DFOV 431.0 mm
A
R
L
kV 140.0
mA: 375
2.0 mm
Tilt: 0.0 degrees
ms
134420.971001
W=200,L=40
10mm/div
P
Brilliance 16P
Ex: 78655
Se: 4
Im: 278
DFOV 431.0 mm
A
R
L
kV 140.0
mA: 375
2.0 mm
Tilt: 0.0 degrees
ms
134421.004000
W=200,L=40
10mm/div
P
Brilliance 16P
Ex: 78655
Se: 4
Im: 279
DFOV 431.0 mm
A
R
L
kV 140.0
mA: 375
2.0 mm
Tilt: 0.0 degrees
ms
134421.038000
W=200,L=40
10mm/div
P
Brilliance 16P
Ex: 78655
Se: 4
Im: 280
DFOV 431.0 mm
A
R
L
kV 140.0
mA: 375
2.0 mm
Tilt: 0.0 degrees
ms
134421.072001
W=200,L=40
10mm/div
P

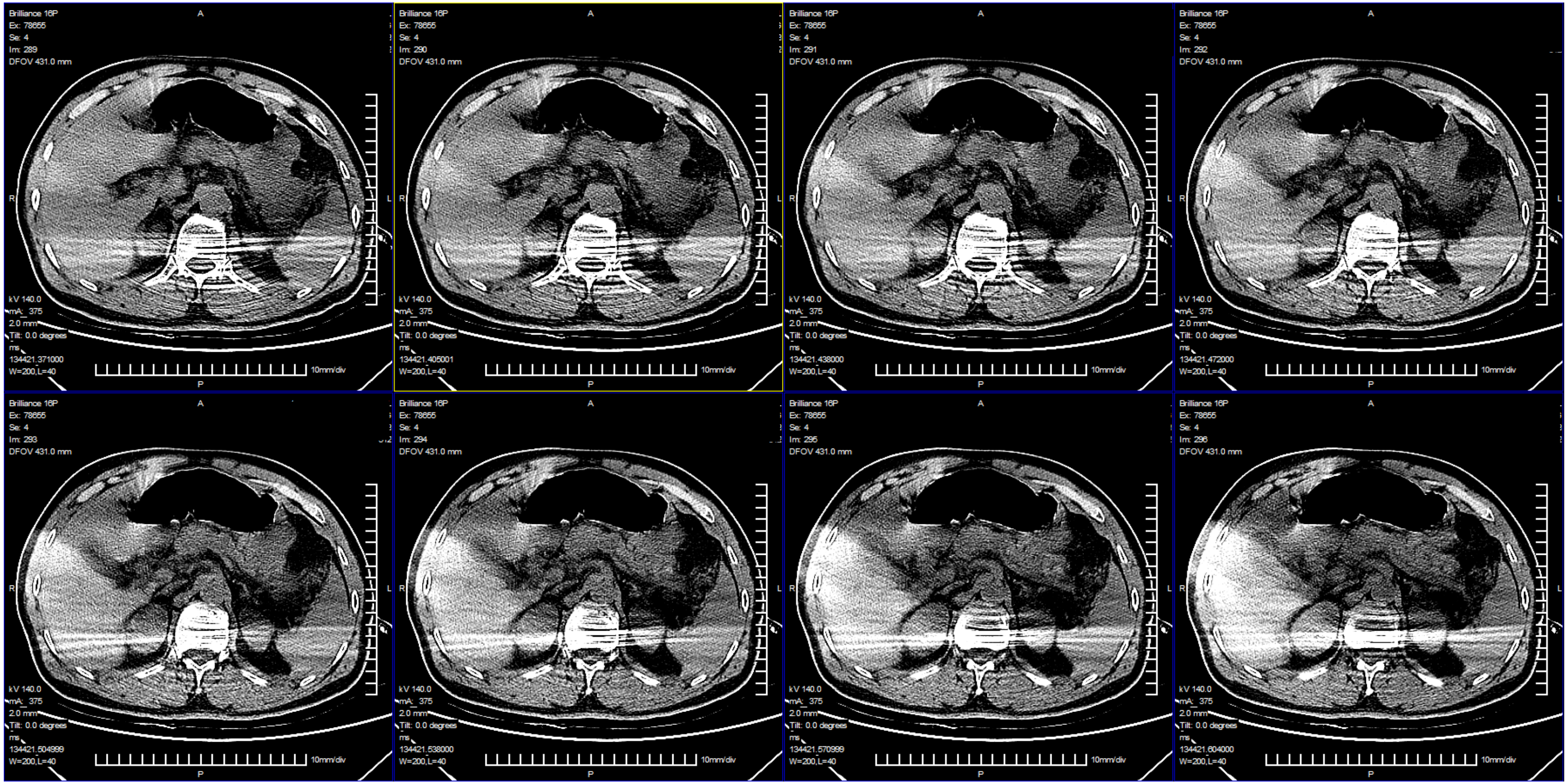
Brilliance 16P
Ex: 78855
Se: 4
Im: 289
DFOV 431.0 mm
A
R
L
kV 140.0
mA: 375
2.0 mm
Tilt: 0.0 degrees
ms
134421.371000
W=200,L=40
10mm/div
P
Brilliance 16P
Ex: 78855
Se: 4
Im: 290
DFOV 431.0 mm
kV 140.0
mA: 375
2.0 mm
Tilt: 0.0 degrees
ms
134421.405001
W=200,L=40
Brilliance 16P
Ex: 78855
Se: 4
Im: 291
DFOV 431.0 mm
kV 140.0
mA: 375
2.0 mm
Tilt: 0.0 degrees
ms
134421.438000
W=200,L=40
Brilliance 16P
Ex: 78855
Se: 4
Im: 292
DFOV 431.0 mm
kV 140.0
mA: 375
2.0 mm
Tilt: 0.0 degrees
ms
134421.472000
W=200,L=40
Brilliance 16P
Ex: 78855
Se: 4
Im: 293
DFOV 431.0 mm
kV 140.0
mA: 375
2.0 mm
Tilt: 0.0 degrees
ms
134421.504999
W=200,L=40
Brilliance 16P
Ex: 78855
Se: 4
Im: 294
DFOV 431.0 mm
kV 140.0
mA: 375
2.0 mm
Tilt: 0.0 degrees
ms
134421.538000
W=200,L=40
Brilliance 16P
Ex: 78855
Se: 4
Im: 295
DFOV 431.0 mm
kV 140.0
mA: 375
2.0 mm
Tilt: 0.0 degrees
ms
134421.570999
W=200,L=40
Brilliance 16P
Ex: 78855
Se: 4
Im: 296
DFOV 431.0 mm
kV 140.0
mA: 375
2.0 mm
Tilt: 0.0 degrees
ms
134421.604000
W=200,L=40

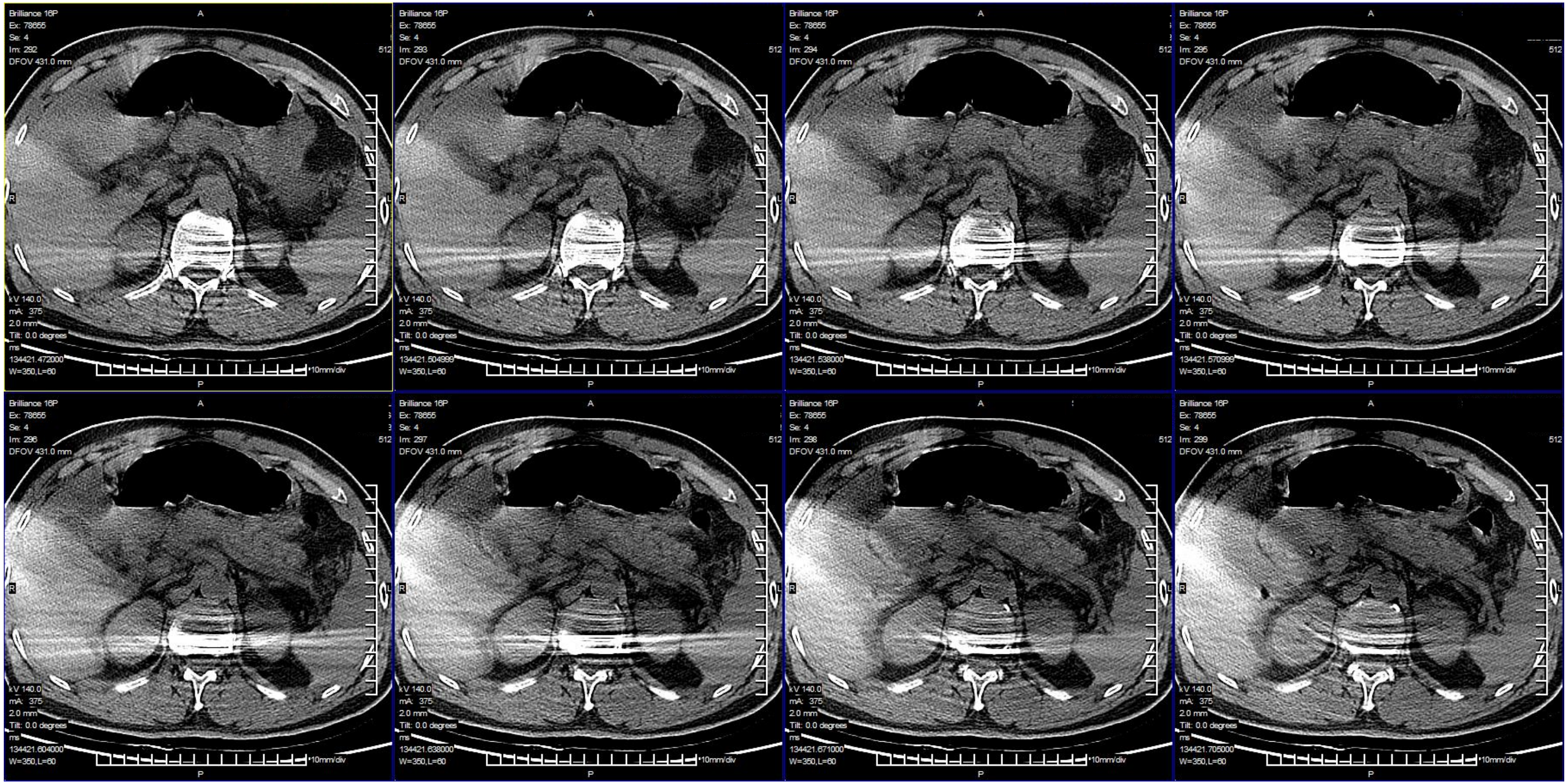
Brilliance 16P
Ex: 78655
Se: 4
Im: 292
DFOV 431.0 mm
A
512
R
L
kV 140.0
mA: 375
2.0 mm
Tilt: 0.0 degrees
ms
134421.472000
W=350,L=60
10mm/div
P
Im: 293
134421.504999
Im: 294
134421.538000
Im: 295
134421.570999
Im: 296
134421.604000
Im: 297
134421.638000
Im: 298
134421.671000
Im: 299
134421.705000

病区: 床号: 日期:2021-02-23 12:35:14

姓名: ID 号: [illegible]55E57F QT/QTc:358/452ms 心房率:96bpm
性别: 住院号: QRS:100ms QRS电轴:-57°
年龄: P-R:156ms RV5/SV1:0.24/0.66mV 心室率:96bpm

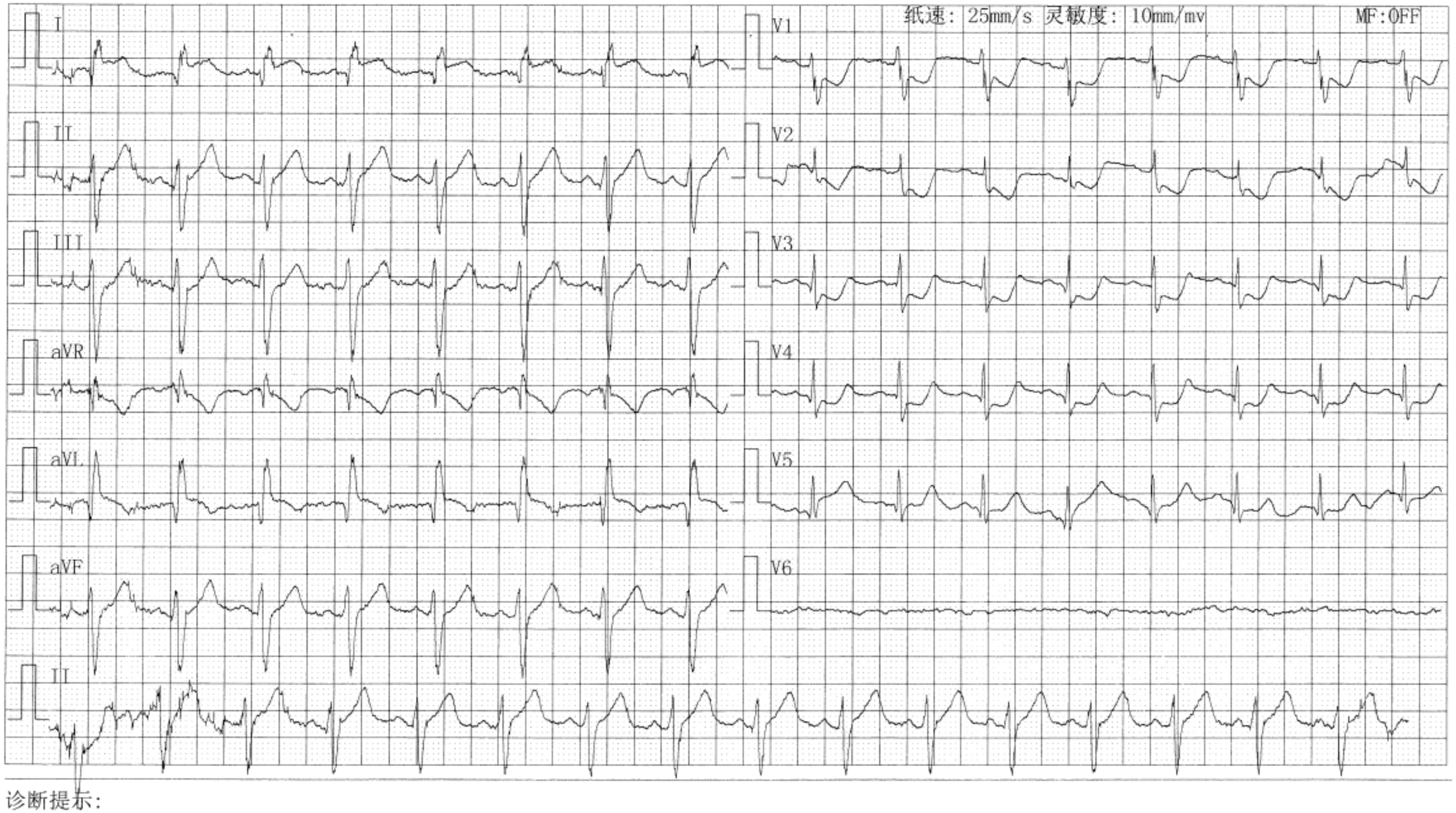

诊断提示:

1、窦性心律
2、V1～V4ST段压低 Ⅰ aVL异常Q波ST段抬高 提示急性高侧壁心梗
3、左前分支阻滞

病区: 床号: 日期:2021-02-23 17:49:17

姓名: ID 号:

性别: 住院号:

年龄:

QRS:80ms

P-R:148ms

QT/QTc:304/415ms

QRS电轴:-1°

RV5/SV1:1.66/0.00mV

心房率:112bpm

心室率:112bpm

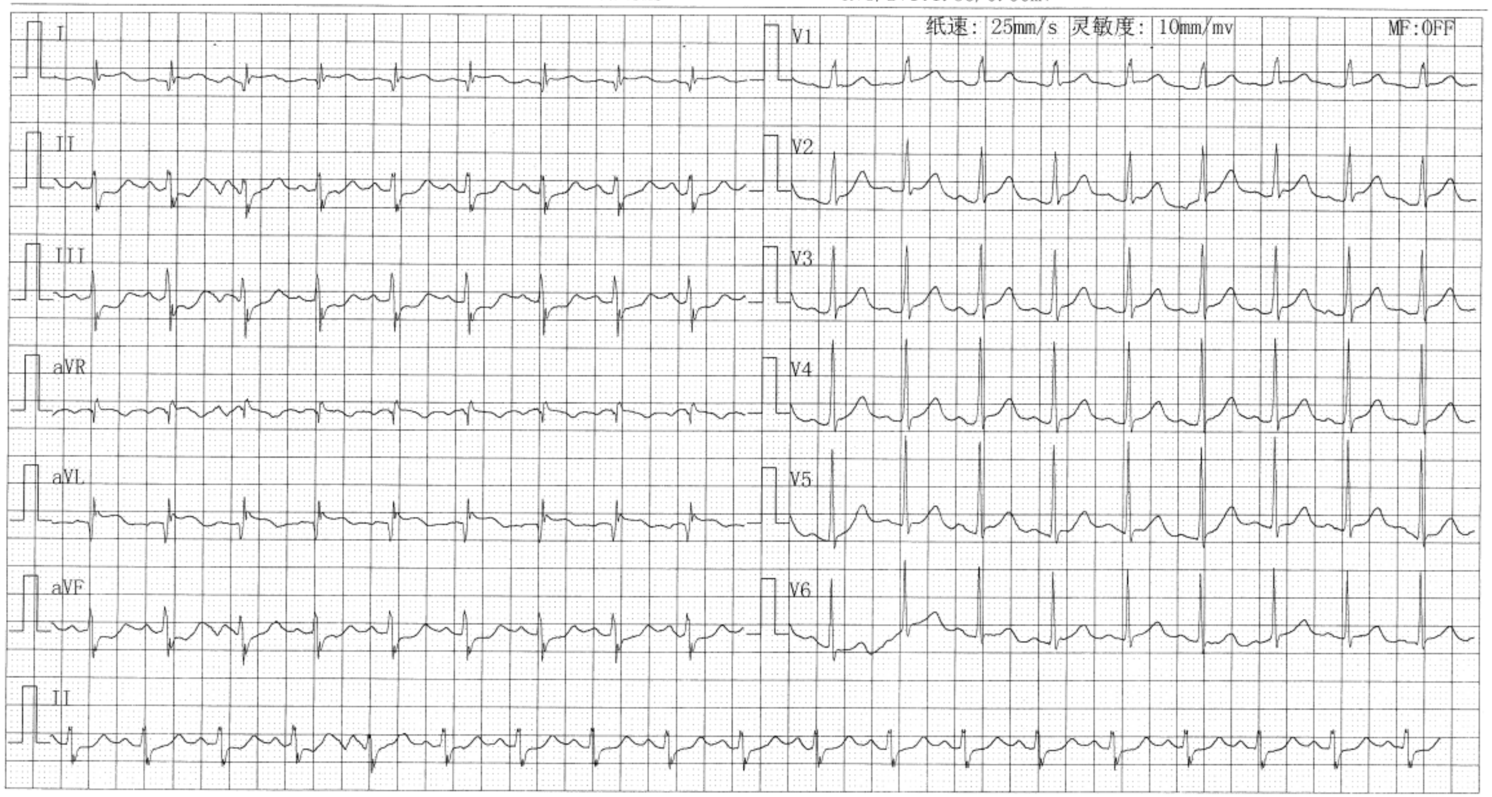

诊断提示:

1、窦性心动过速

2、逆钟向转位

3、 Ⅰ aVL异常Q波ST段抬高 提示急性高侧壁心梗

4、ST段压低ⅡⅢaVF

日期:2021-02-24 09:54:21

姓名:　　　　　　　　ID　号:
性别:　　　　　　　　住院号:
年龄:

P-R:164ms

QT/QTc:338/409ms
QRS电轴:-90°
RV5/SV1:0.00/0.00mV

心房率:88bpm
心室率:88bpm

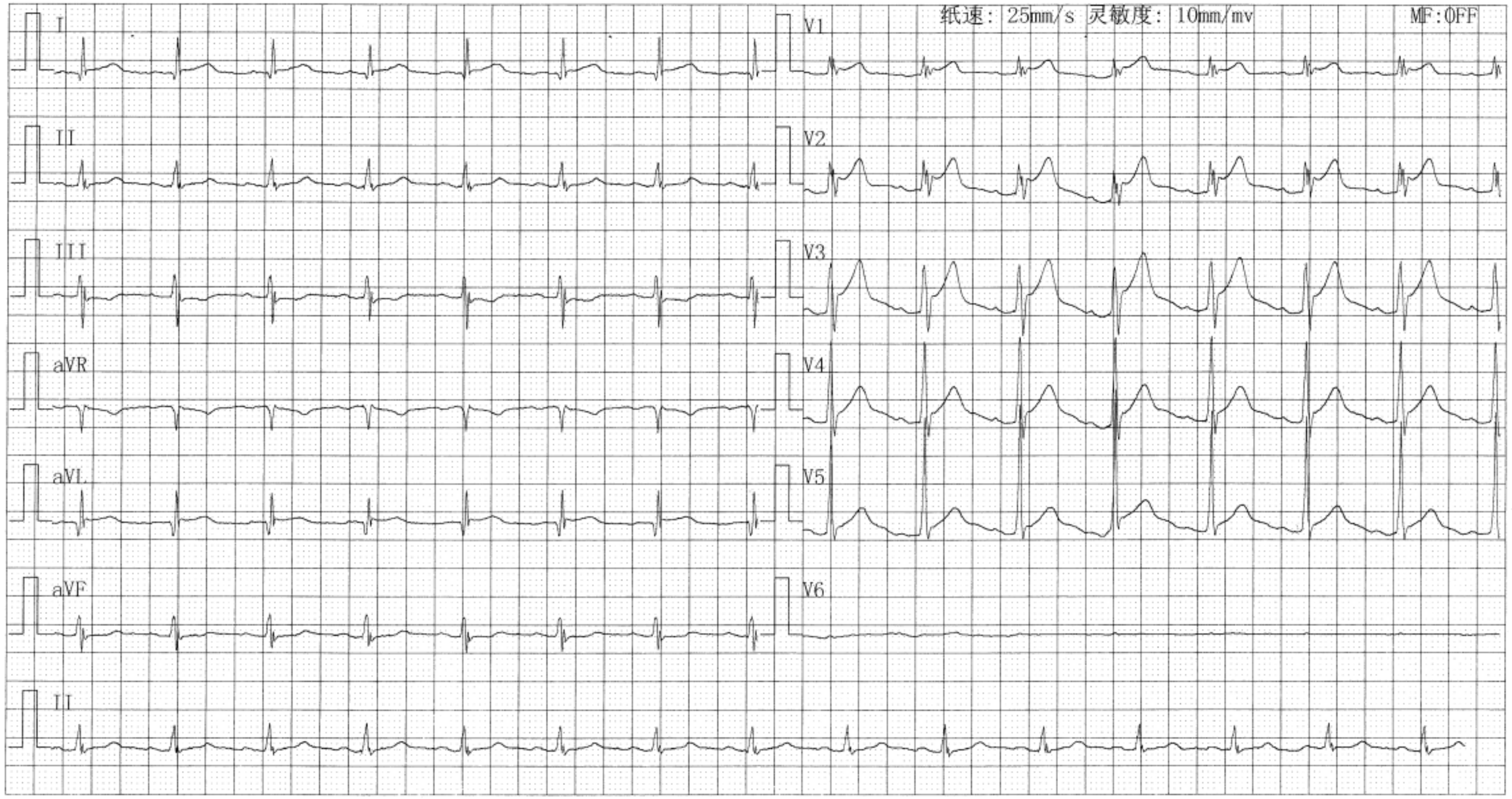

诊断提示:

1、窦性心律
2、V1～V3ST段抬高 提示：急性前间壁心肌梗死
3、ⅠaVLQ波异常 提示：急性高侧壁心肌梗死

[illegible]

日期:2021-02-25 11:50:10

姓名:	[illegible]F55E57F	QT/QTc:330/430ms	心房率:102bpm
性别:	QRS:92ms	QRS电轴:+22°	
年龄:	P-R:142ms	RV5/SV1:2.10/0.19mV	心室率:102bpm

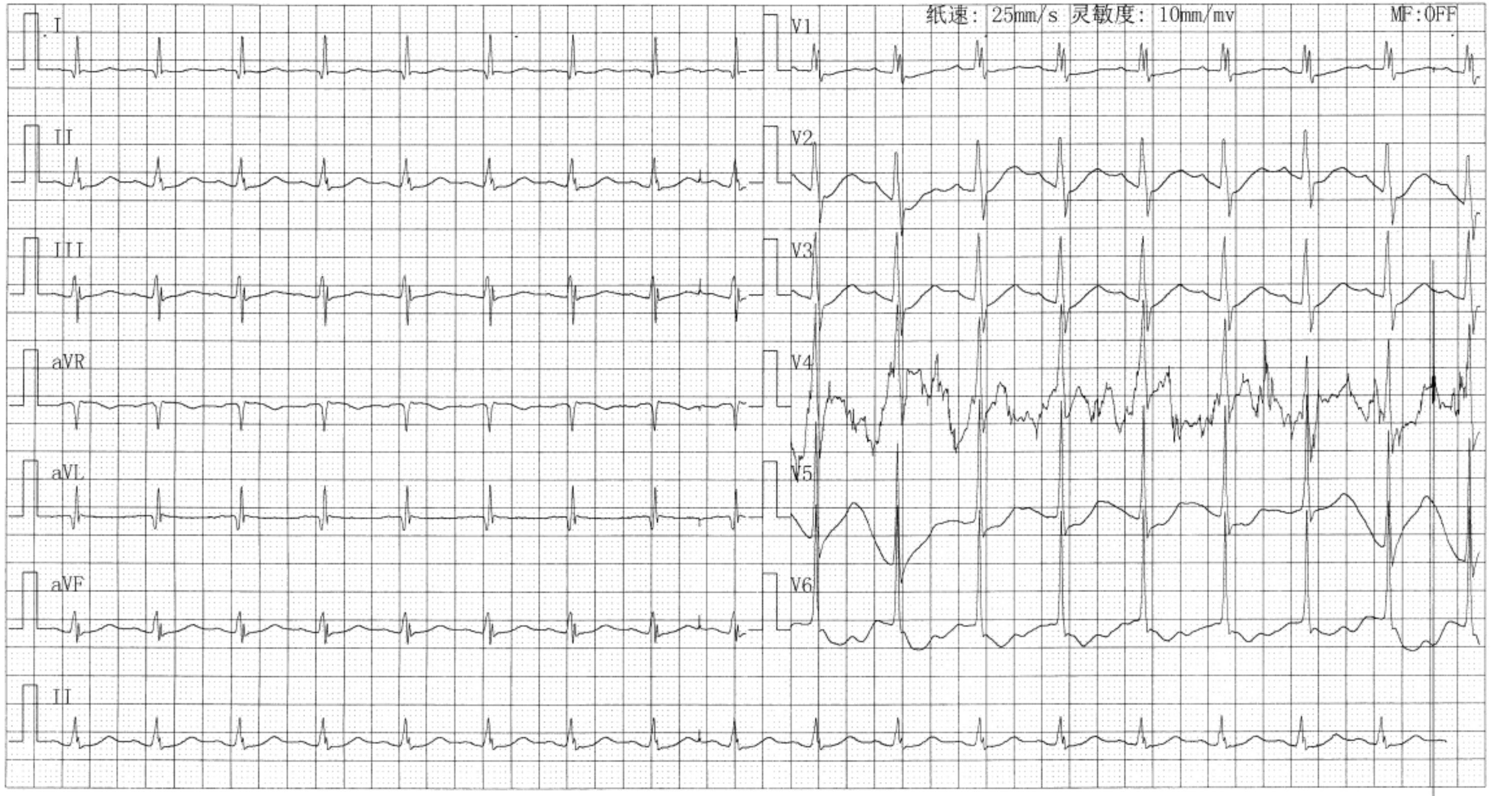

诊断提示:

1、窦性心动过速
2、Ⅰ、aVL可见Q波，请结合临床
3、ST-T改变（V5、V6）

日期:2021-02-26 11:38:36

姓名:
性别:
年龄:

QT/QTc:366/435ms
QRS电轴:+34°
RV5/SV1:0.00/0.17mV

心房率:85bpm
心室率:85bpm

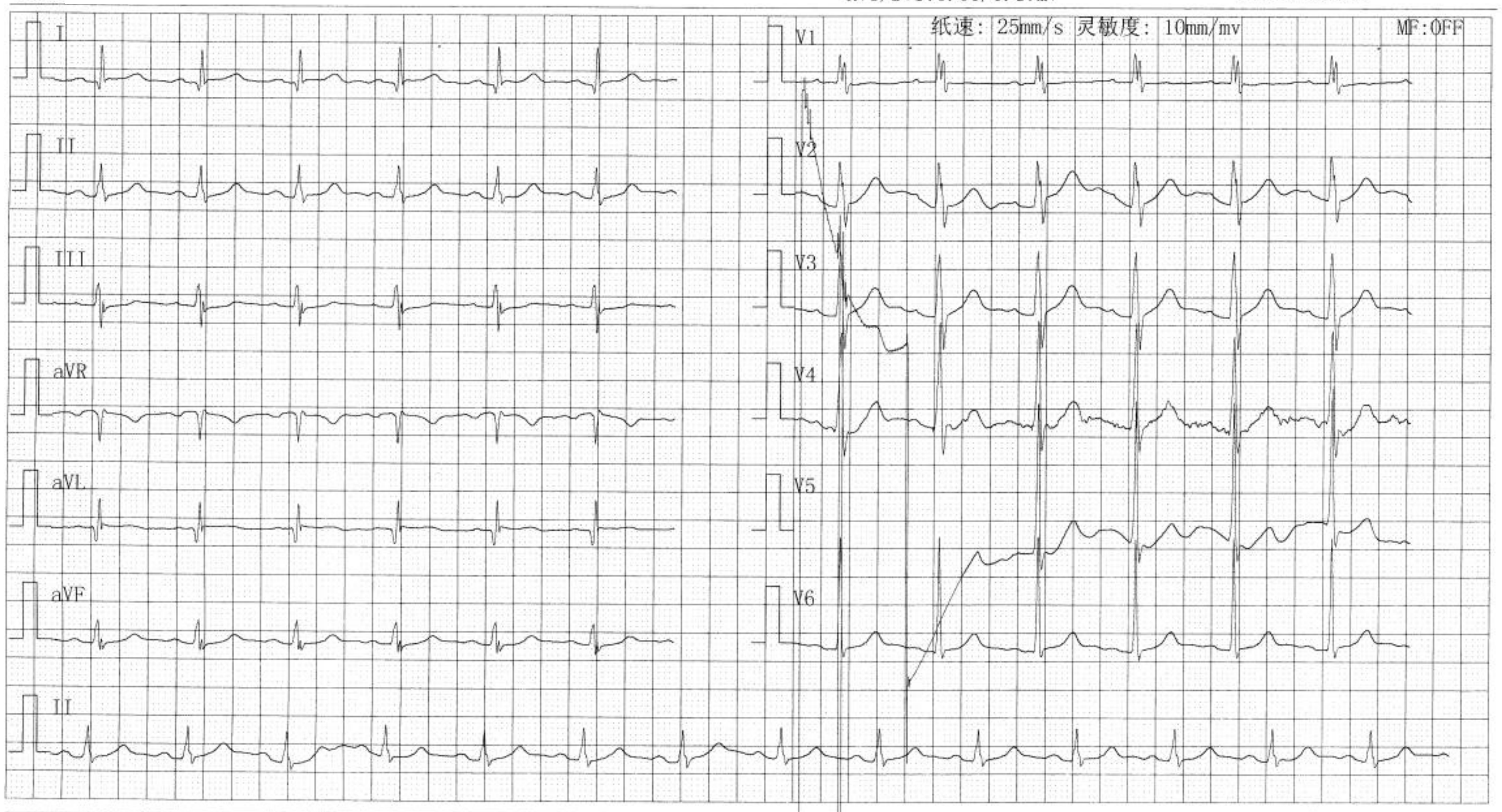

诊断提示:

1、窦性心律
2、逆钟向转位
3、Q波异常 I aVL

病区: 床号: 日期:2021-03-02 18:06:13

姓名: ID 号:
性别: 住院号:
年龄:

QRS:82ms
P-R:150ms

QT/QTc:360/448ms
QRS电轴:+55°
RV5/SV1:3.45/0.14mV

心房率:93bpm
心室率:93bpm

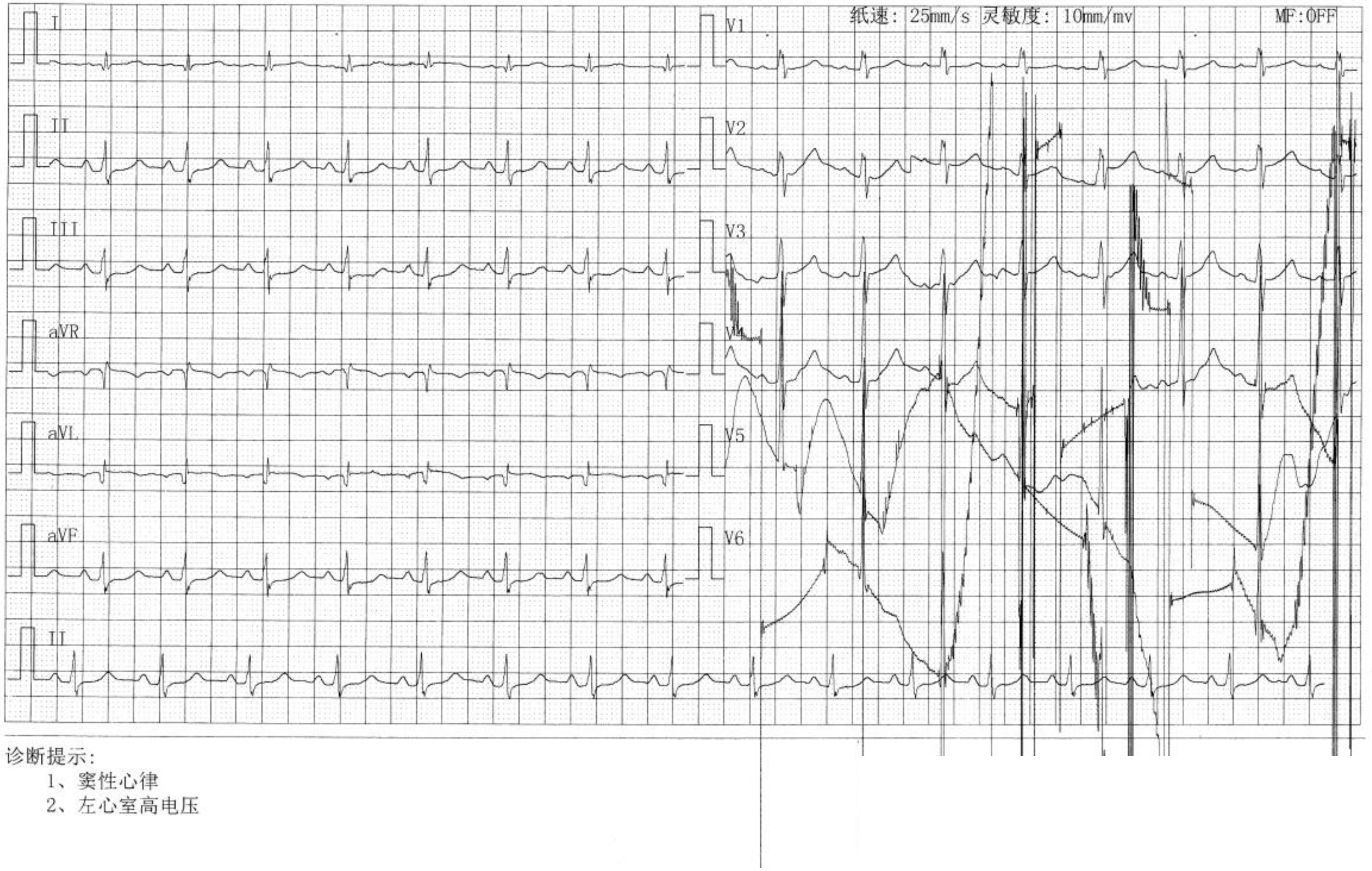

诊断提示:

1、窦性心律
2、左心室高电压

日期:2021-03-03 15:17:58

QT/QTc:292/384ms
QRS电轴:+98°
RV5/SV1:1.20/0.00mV

心房率:104bpm
心室率:104bpm

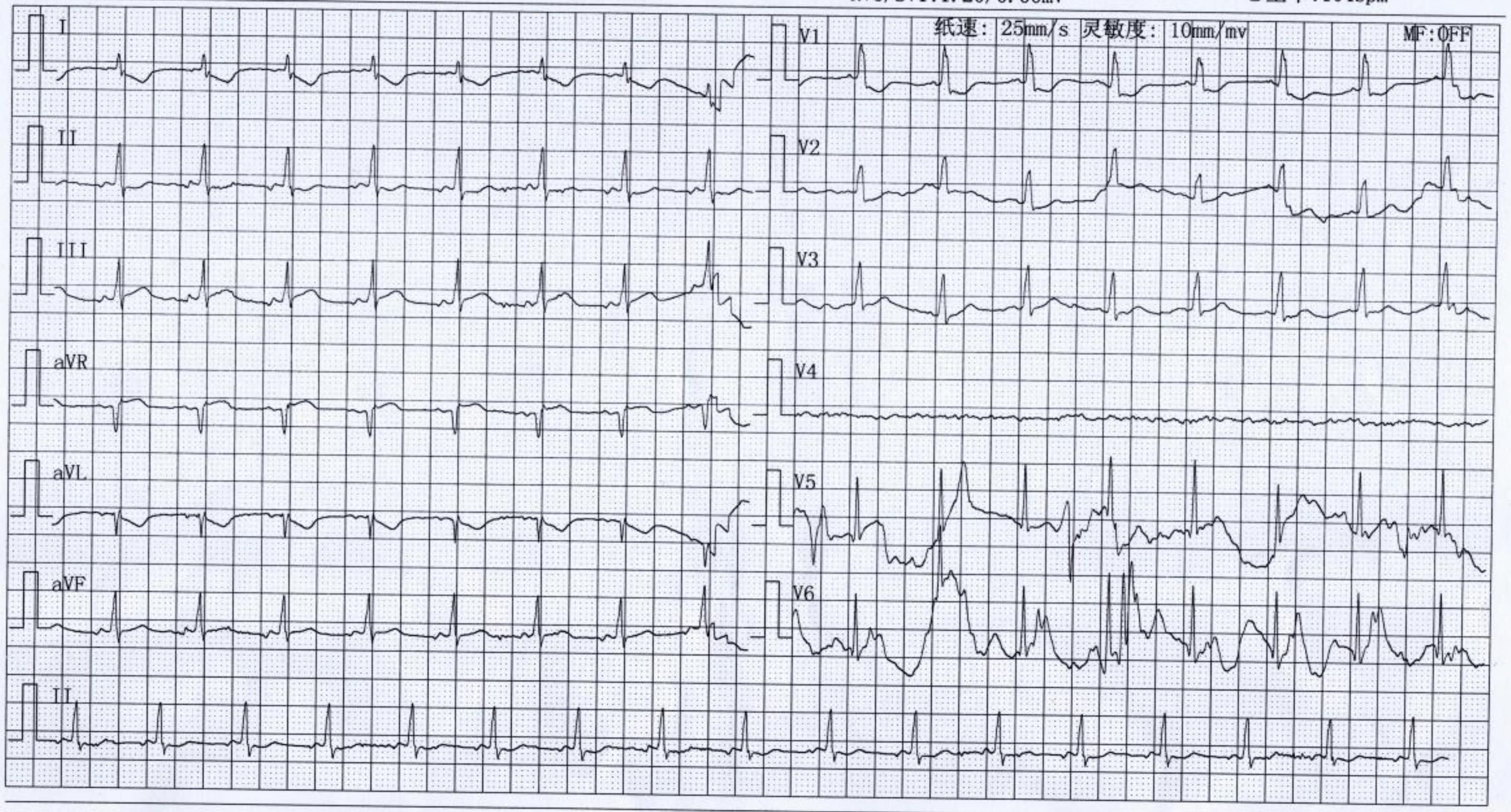

诊断提示:

1、窦性心动过速
2、ST-T改变 I aVL
3、逆钟向转位
