# Supplementary figures and images for: Case report: Delayed cardiac rupture with congenital absence of pericardium after blunt trauma
Source: Front Cardiovasc Med. 2022 Dec 20;9:1079670. doi: 10.3389/fcvm.2022.1079670 (PMC9808968; doi:10.3389/fcvm.2022.1079670)

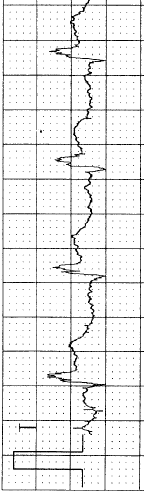

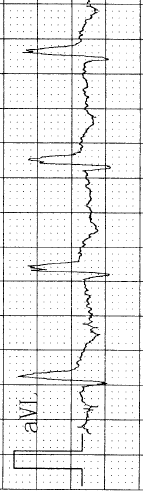


2021-02-23 12:00


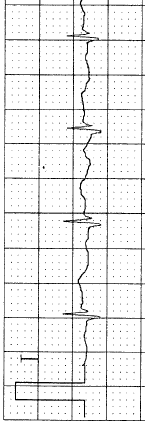

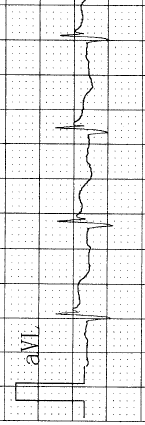


2021-02-23 17:00


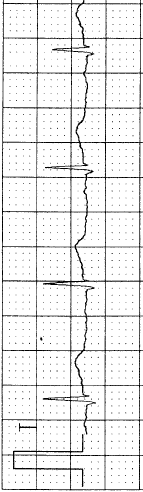

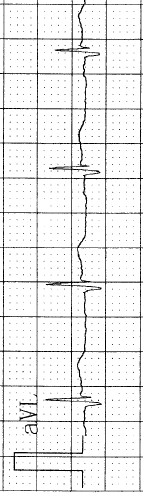


2021-02-24


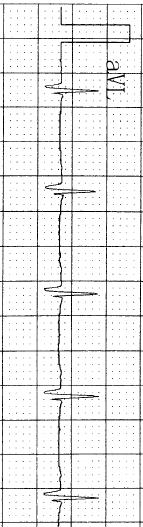

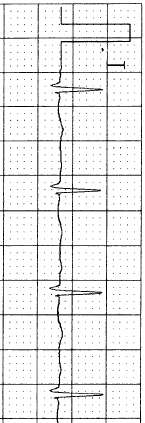


2021-02-25


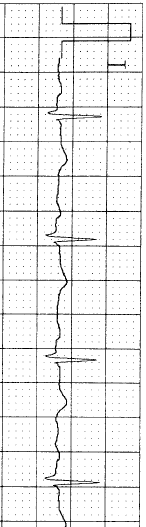

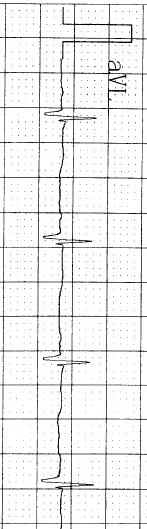


2021-02-26


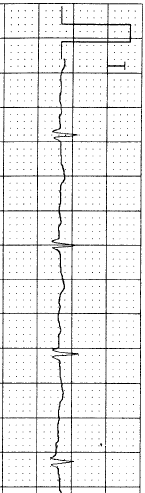

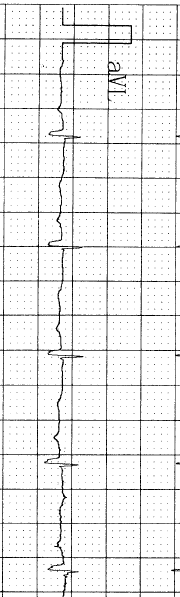


2021-03-02


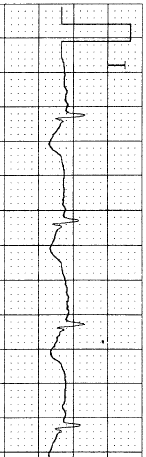

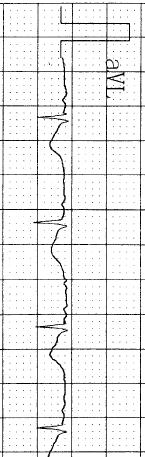


2021-03-03

Supplement: Supplementary file 2 [file Table_1.DOCX]
